# The natural *Disc1*-deletion present in several inbred mouse strains does not affect sleep

**DOI:** 10.1038/s41598-017-06015-3

**Published:** 2017-07-18

**Authors:** Lars Dittrich, Alessandro Petese, Walker S. Jackson

**Affiliations:** Deutsches Zentrum für Neurodegenerative Erkrankungen (DZNE), Sigmund-Freud-Str, 27 53127 Bonn, Germany

## Abstract

The gene Disrupted in Schizophrenia-1 (*DISC1*) is linked to a range of psychiatric disorders. Two recent transgenic studies suggest *DISC1* is also involved in homeostatic sleep regulation. Several strains of inbred mice commonly used for genome manipulation experiments, including several Swiss and likely all 129 substrains, carry a natural deletion mutation of *Disc1*. This constitutes a potential confound for studying sleep in genetically modified mice. Since disturbed sleep can also influence psychiatric and neurodegenerative disease models, this putative confound might affect a wide range of studies in several fields. Therefore, we asked to what extent the natural *Disc1* deletion affects sleep. To this end, we first compared sleep and electroencephalogram (EEG) phenotypes of 129S4 mice carrying the *Disc1* deletion and C57BL/6N mice carrying the full-length version. We then bred *Disc1* from C57BL/6N into the 129S4 background, resulting in S4-Disc1 mice. The differences between 129S4 and C57BL/6N were not detected in the 129S4 to S4-Disc1 comparison. We conclude that the mutation has no effect on the measured sleep and EEG characteristics. Thus, it is unlikely the widespread *Disc1* deletion has led to spurious results in previous sleep studies or that it alters sleep in mouse models of psychiatric or neurodegenerative diseases.

## Introduction

Many psychiatric disorders, including schizophrenia, major depression, and bipolar disorder, are accompanied by pronounced sleep abnormalities^1, 2^. This was long viewed as a secondary effect downstream of the primary disorder. However, recent evidence suggests that the relation is bidirectional, i.e. sleep problems exacerbate psychiatric diseases^3–6^. The cause for the high rate of co-occurrence of psychiatric disorders and sleep abnormalities is unknown, but a common genetic predisposition might increase the risk for both^3–5, 7, 8^. A Scottish pedigree with high prevalence of psychiatric disease carries a mutated gene associated with schizophrenia, major depression and bipolar disorder^9^. The gene, known as Disrupted in Schizophrenia-1 (*DISC1*) encodes an intracellular scaffold protein known to play a role in neuronal development and synaptic function^10^. A role for *Disc1* in sleep regulation was suggested in two transgenic studies ectopically expressing human *DISC1*. In flies human *DISC1* caused increased total sleep and increased sleep bout duration whereas in mice it caused increased wakefulness and decreased sleep^11, 12^. Although these two reports seem contradictory, it should be noted that flies lack a native *Disc1* gene and the transgenic mice expressed both human and mouse *Disc1* and that the measurement techniques for flies and mice are necessarily different. Nonetheless, these reports indicate that expression of *DISC1* related genes can alter sleep.

In contrast to most inbred mouse strains such as C57BL/6, several strains including “Swiss” strains (e.g. FVB, SJL, SWR), and all 129 substrains naturally carry a 25 base pair deletion mutation in exon 6 of *Disc1* ^13–15^. Importantly, many of these strains have been widely used for genome manipulation techniques. The molecular consequence of the deletion is that the normal Disc1 protein can be made up to amino acid 528, after which a stretch of 13 incorrect amino acids are added until a newly in-frame stop codon is reached and translation is terminated. Since full-length wild-type Disc1 protein has 852 amino acids, the deletion allele theoretically removes 38% of the C-terminal end of Disc1 protein and replaces it with 13 amino acids of unknown consequence. It is conceivable that this mutant form loses all or most of the normal function or gains a negative function. Importantly, although western blot analysis confirmed that the corresponding region of Disc1 protein is not detected in brains of mice homozygous for the *Disc1* deletion, antibodies specific for peptides on either side of the deleted region indicate that a nearly full-length Disc1 protein is expressed, most likely the result of alternative splicing or exon skipping^16^. Despite synthesizing much of the protein, mice congenic for the *Disc1* deletion show abnormalities in behavioral tests, pre-pulse inhibition, neuron morphology, and neuronal excitability^17–19^. Thus, the full significance of the deletion remains uncertain.

Considering the above mentioned reports of altered sleep in human *DISC1* expressing transgenic flies and mice and that the mouse *Disc1* deletion creates physiological changes, we wondered if the *Disc1* deletion would also affect sleep. This is important both for the quest of understanding links between sleep and psychiatric disease and to determine if it creates a potential confound for a large number of existing studies. A search on the Jackson laboratories website suggests that in their collection alone over 4,000 mouse lines have some contribution of a 129 genome, and over 1,000 of FVB (as of February 2017). Considering that sleep influences several non-sleep measures in mice, such as psychiatric behaviors^8^, memory^20^, and neurodegeneration^21^, this confound could affect a wide range of published mouse studies. To test if the *Disc1* deletion does affect sleep, we characterized the sleep phenotype of a commonly used substrain, 129S4 (formerly 129/SvJae, hereafter S4). To our knowledge, the sleep phenotype of this substrain has never been characterized, despite its wide use. Sleep has been well characterized in the 129P2 substrain formerly 129/Ola^22, 23^. However, 129 substrains are highly diverse genetically^24, 25^, which cautions against transferring phenotyping results between substrains. We identified several differences in sleep-related characteristics between S4 and C57BL/6N (hereafter B6N). We then asked whether any of these differences were caused by the *Disc1* deletion. To this end we bred the full length B6N version of the *Disc1* gene to the S4 background yielding a mouse line that carries the *Disc1* locus from B6N in the context of a > 99.6% S4 genome, a line we call S4-Disc1. We reasoned that any sleep characteristic that was disrupted by the *Disc1* deletion should resemble the B6 phenotype and differ from the S4 phenotype in these mice. Surprisingly, we detected no differences between S4 and S4-Disc1 mice.

## Results

**Figure 1:**
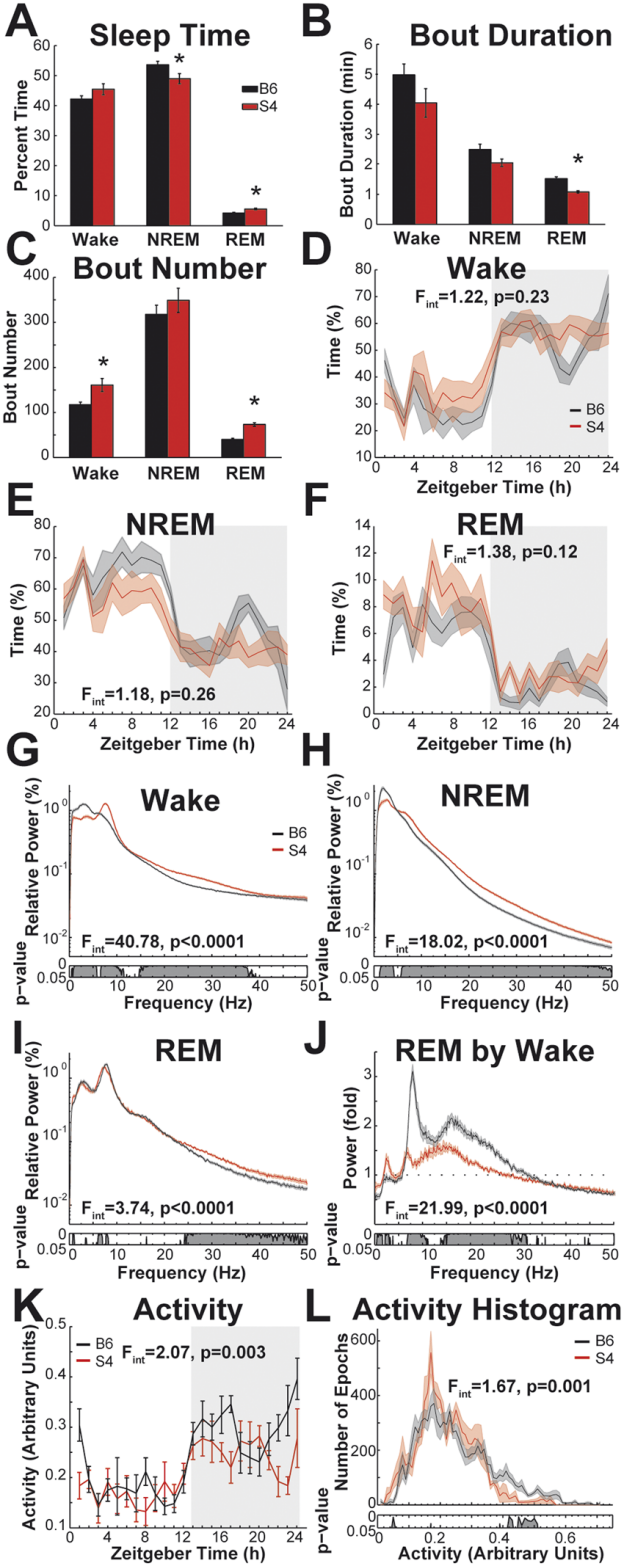
Vigilance states of S4 and B6 mice during 24 h undisturbed baseline. (**A**) percentage of time spent in wake, NREM, and REM. Asterisks indicate differences between strains at p < 0.05. (**B**) Average durations of the bouts of each state. (**C**) Average number of bouts during baseline. (**D**) Wake time across the 24 h in 1 h bins. Curves depict group averages, surrounding shaded areas depict s.e.m. Shaded rectangle indicates time during lights off. F-values for interactions of factors ‘time’ and ‘strain’ are indicated in the panels. Degrees of freedom for ANOVAs in D-F are 23, 276. (**E**) NREM time across the 24 h (**F**) REM time across the 24 h. (**G**–**I**) EEG power spectra. The power in one frequency bin is expressed as percentage of the cumulative power of all frequencies (0–50 Hz). Curves depict group averages, shaded areas depict s.e.m. Interactions of factors ‘frequency’ and ‘strain’ (permutation ANOVA) are indicated for each panel. The degrees of freedom are 409 and 4908 for all interactions in **G**–**J**. The p-values for post hoc uncorrected bin-by-bin t-tests are indicated in grey below the spectra (**G**) Average wake power spectra (**H**) Average NREM EEG power spectra. (**I**) Average REM EEG power spectra. (**J**) Average REM EEG power spectra normalized by the respective wake power spectra. (**K**) Average activity during baseline in 1 h bins. Error bars depict s.e.m. F-value for interaction of factors ‘time’ and ‘strain’ is indicated in the panel. Degrees of freedom are 23, 276. (**L**) Frequency time histogram (71 bins) of activity during baseline. Curves depict group averages, shaded areas depict s.e.m. F-value for interaction of factors ‘activity level’ and ‘strain’ (permutation ANOVA) is indicated in the panel. Degrees of freedom are 70, 840. P-values of non-corrected post-hoc t-tests (bottom) indicate that B6 mice spent more time at activity levels > 0.4.

Vigilance states can be classified into wake, REM (rapid eye movement sleep) and NREM (non-rapid eye movement sleep). To compare the sleep phenotype of S4 mice to B6N mice, EEG and EMG (electromyogram) were recorded using wireless implanted transmitters that allowed unrestricted movement in the cage. We manually scored vigilance states in 10-second epochs. We then tested if the distribution or architecture of these states differed across an undisturbed 24 h baseline period. During baseline, S4 mice spent less time in NREM (T_12_ = 2.33, p = 0.038) and more time in REM (T_12_ = 3.59, p = 0.004) than B6 mice (Fig. 1A). Group means with standard deviations were 45.5 ± 1.8% wake, 49.0 ± 1.7% NREM, 5.5 ± 0.3% REM for S4 and 42.2 ± 1.1% wake, 53.6 ± 1.2% NREM, 4.2 ± 0.2% REM for B6N. Average REM bout duration was shorter (T_12_ = 5.67, p < 0.001), whereas the number of REM (T_12_ = 6.79, p < 0.001) as well as wake (T_12_ = 3.08, p = 0.009) bouts was higher (Fig. 1B and C). Group means for bout durations were 4.0 ± 0.5 min wake, 2.1 ± 0.1 min NREM, 1.1 ± 0.04 min REM for S4, 5.0 ± 0.4 min wake, 2.5 ± 0.2 min NREM, 1.5 ± 0.06 min REM for B6N. Group means for bout numbers were 160.8 ± 14.5 wake, 349.2 ± 26.9 NREM, 73.3 ± 3.9 REM for S4, 117.4 ± 5.8 wake 317.9 ± 20.4 NREM, 39.9 ± 3.1 REM for B6N. The distribution of sleep time across the 24 h period illustrates that differences in sleep amount were not apparently restricted to certain times of day (Fig. 1D–F; no interactions of factors ‘Zeitgeber Time’ and ‘strain’).

To test if the S4 EEG differs from that of B6N mice, we calculated the average EEG power spectra. This analysis quantifies the relative contribution of different frequencies to the EEG and thus enables the detection of differences across a wide range of frequencies. EEG strongly varies with vigilance state, which can compromise EEG analyses if sleep/wake behavior is not considered. Therefore, power spectra were analyzed for each of the three vigilance states separately. During wake, S4 mice produced lower relative power in the delta range (1–4 Hz) and higher relative power in the theta range (4–10 Hz) and in frequencies between 15 and 40 Hz (Fig. 1G). During NREM, S4 mice produced lower relative power in the delta range and higher power in all higher frequencies (Fig. 1H). During REM, the relative power at frequencies > 25 Hz was higher in S4 (Fig. 1I). The average theta shifted towards lower frequencies in S4 mice, resulting in higher power at 6–7 Hz and lower power at 7.5–8 Hz. Expressing power spectra as relative values is a useful way for normalization when comparing recordings from different individuals. However, it can be difficult to discern if an observed difference is driven by higher absolute power in one frequency range or lower absolute power in another one. Therefore, it can be useful to normalize the data additionally in a different, non-redundant way. To this end we plotted the REM spectra normalized by the corresponding wake spectra (Fig. 1J). The difference between REM and wake was smaller in the S4 than in the B6N mice, because of their somewhat REM-like wake spectra with a large theta peak and high relative power in the 10–30 Hz range. Thus, the strains differed not just in EEG composition, but also in the way the EEG changed when transitioning between vigilance states. The differences in EEG spectra between B6 and S4 were so pronounced that they were also discernible in non-normalized raw spectra (Supplementary Fig. 1A–C).

To test if the mouse strains showed overall differences in locomotor activity, we made use of a semi-quantitative measure based on changes of signal strength when the transmitter moves relative to the receiver antennae. S4 mice showed on average lower locomotor activity. Analysis of diurnal activity patterns in hourly bins (Fig. 1K) revealed main effects for the factors ‘strain’ (F_1,12_ = 6.05, p = 0.03) and ‘Zeitgeber Time’ (_F23,276_ = 7.23, p < 0.001) as well as their interaction (F_23,276_ = 2.07, p = 0.003). Correspondingly, Fig. 1K indicates that differences in average locomotor activity were most pronounced during lights-off. For a more detailed analysis, we plotted the activity recordings from all 10 s-epochs as histograms (Fig. 1L). This kind of analysis compares the distribution of activity levels, which can reveal more subtle information than comparison of means. For example, lower average activity could be caused by a generally slower locomotion, but just as well by more time spent without movement or a lower number of bursts of very high activity levels. We found that S4 mice spent less time at the highest activity levels of values > 0.4 (statistics in Fig. 1L). Analysis of the “activity count” measure provided by the recording software confirmed that S4 showed lower locomotor activity than B6 (Supplemental Fig. 1D–F).

**Figure 2:**
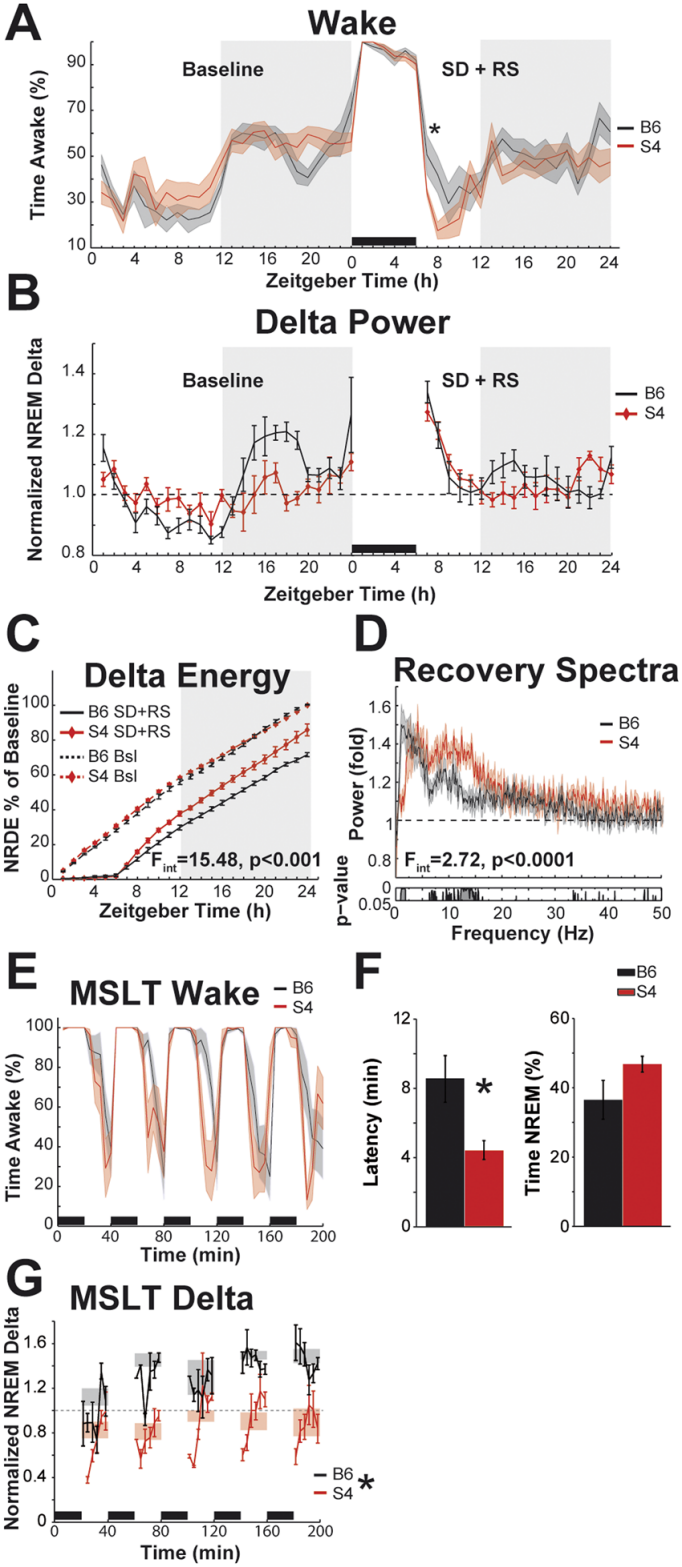
Responses to sleep homeostatic challenges in S4 and B6 mice. (**A**) Wakefulness in hourly bins during undisturbed baseline, sleep deprivation, and recovery opportunity. Black bar at bottom indicates sleep deprivation. Shaded background indicates lights-off. Curves depict group means and s.e.m. The baseline values are replotted from Fig. 1D. The asterisk indicates the significant difference between strains in sleep amounts during the first 2 h of recovery sleep opportunity (p = 0.02). SD = sleep deprivation, RS = recovery sleep (**B**) NREM delta power in hourly bins during baseline and recovery sleep opportunity. Black bar at bottom indicates sleep deprivation. Shaded background indicates lights-off. Curves depict group means and s.e.m. The amplitude of oscillation was lower in S4 mice. (**C**) Cumulative NREM delta energy over 24 h. Dashed curves depict baseline values, solid curves depict values during sleep deprivation and recovery sleep. Curves depict mean values and s.e.m. F-value for interaction of factors ‘time’ and ‘strain’ for the sleep deprivation + recovery days is indicated in the panel. Degrees of freedom are 23, 276. Bsl = baseline. (**D**) NREM EEG power spectra of the first 2 h of RS normalized by the corresponding 2 h of baseline NREM (ZT7 + 8). Curves depict group averages and s.e.m. Interaction of factors ‘frequency’ and ‘strain’ (permutation ANOVA) is indicated at the bottom. The degrees of freedom are 409 and 4908. The p-values for post hoc uncorrected bin-by-bin t-tests are indicated below the spectra. (**E**) Wake time during the multiple sleep latency test. Curves depict group means and s.e.m. Black bars at bottom indicate the five consecutive 20 min sleep deprivations. (**F**) Sleep latency and time spent in NREM during the 5 nap opportunities of the MSLT. Asterisk indicates shorter sleep latencies for S4 mice (p < 0.05). (**G**) NREM delta power during the nap opportunities of the MSLT, normalized by the average baseline values. Curves depict group means in 200 s time bins. Error bars depict s.e.m. Shaded rectangles depict group means ± s.e.m. for a complete nap opportunity. The asterisk by the legend indicates a significant main effect of ANOVA for the factor ‘strain’ (p < 0.05).

For a more detailed analysis of sleep regulation, we subjected the mice to a sleep homeostatic challenge. Mice were kept awake for 6 h, after which they were left undisturbed, providing an opportunity for recovery sleep. In the first 2 h of RS opportunity following sleep deprivation, S4 mice spent more time asleep than B6N mice (74.0% ± 2.5 and 53.7% ± 6.5, respectively; T_12_ = 2.58, p = 0.024; Fig. 2A). An established measure of accrued sleep pressure is the NREM EEG power in the 1–4 Hz range (NREM delta power). Figure 2B depicts NREM delta power during baseline and following 6 h of sleep deprivation, both normalized to the average 24 h baseline values. During baseline, the diurnal oscillation was smaller in S4 than B6N mice (24 h trough-to-peak amplitude of hourly time bins 0.38 ± 0.02 for S4, 0.61 ± 0.08 for B6, T_12_ = 2.32, p = 0.038). The expected compensatory peak of NREM delta power following sleep deprivation did not differ between strains. A measure for sleep homeostasis complementary to NREM delta power is NREM delta energy (NRDE, called Delta energy in SWS by Franken *et al*.^26^). It is the mathematical product of NREM delta power and time spent in NREM, and thus reflects both homeostatic responses to sleep loss, increasing sleep time and increasing sleep depth. Following 6 h of sleep deprivation, S4 mice regained cumulative NREM delta energy quicker than B6N mice (Fig. 2C). Correspondingly, ANOVA revealed an interaction between factors ‘Zeitgeber Time’ and ‘strain’, indicating that the cumulative NRDE curves differ (F_23,276_ = 15.48, p < 0.001). To analyze the EEG response to sleep deprivation in more detail, we plotted the average NREM spectra during the 2 h recovery sleep opportunity normalized by the NREM spectra of the corresponding 2 h of baseline recording, i.e. Zeitgeber Time (ZT) 7–8. This depiction illustrates the changes in NREM EEG spectra in response to sleep deprivation (statistics in figure). S4 mice responded with a weaker increase in low delta power (<2 Hz), and a stronger increase in the frequencies between 7 and 15 Hz than B6N (Fig. 2D).

To further investigate sleep regulation, we subjected both strains to the murine version of the multiple sleep latency test (MSLT)^27^, a test that measures sleep propensity and is used similarly in humans. In this test, S4 mice showed a shorter latency to sleep than B6N (4.4 ± 0.5 min and 8.6 ± 1.4 min, respectively; T_12_ = 2.47, p = 0.029), indicating higher sleepiness (Fig. 2E,F). When we analyzed NREM delta power during the nap opportunities in the MSLT, we found that B6N mice responded to that test with a stronger increase in delta power relative to baseline. ANOVA revealed a main effect for ‘strain’ (_F1,9_ = 23.39, p = 0.001) but no main effect of ‘nap opportunity’ or interaction.

To test if any of the differences identified in Figs 1 and 2 might be influenced by the *Disc1* deletion of S4, we compared a second group of S4 mice with S4-Disc1 mice, which carry the B6N version of the gene on an S4 background. The S4-Disc1 line was developed by passing the B6N *Disc1* gene through the S4 background for 9 generations. Single nucleotide polymorphisms (SNPs) are DNA substitutions in coding and non-coding regions distributed throughout the genome and are commonly used as markers to determine the genetic relatedness of different mouse strains. At generation seven, the developing S4-Disc1 line was determined to be 99.58% S4 by a genome wide SNP survey, leading us to conclude that by generation nine it was >99.6% S4.

**Figure 3:**
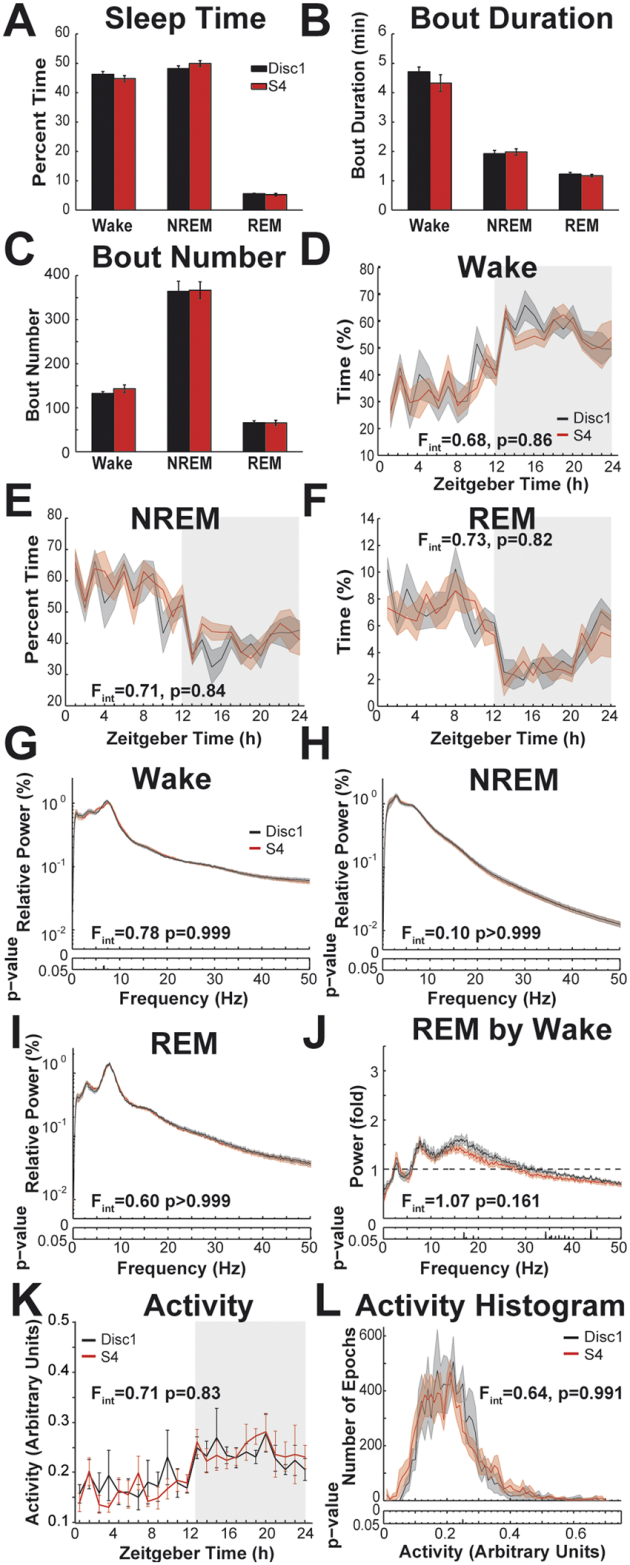
Vigilance states of S4 and S4-Disc1 mice during 24 h undisturbed baseline. This figure directly parallels the comparisons made between S4 and B6 in Fig. 1. (**A**) Percentage of time spent in wake, NREM, and REM. (**B**) Average durations of the bouts of each state. (**C**) Average number of bouts during baseline. (**D**) Wake time across the 24 h in 1 h bins. Curves depict group averages, shaded areas depict s.e.m. Shaded rectangle indicates lights off. F-value﻿s for interactions of factors ‘time’ and ‘strain’ are indicated in the panels. Degrees of freedom for ANOVAs in D-F are 23, 276. (**E**) NREM time across the 24 h. (**F**) REM time across the 24 h. (G-I) EEG power spectra. The power in one frequency bin is expressed as percentage of the cumulative power of all frequencies (0–50 Hz). Curves depict group averages, shaded areas depict s.e.m. Interactions of factors ‘frequency’ and ‘genotype’ (permutation ANOVA) are indicated for each panel. The degrees of freedom are 409 and 4908 for interactions in G-J. The p-values for post hoc uncorrected bin-by-bin t-tests are indicated below the spectra (**G**) Average wake power spectra (**H**) Average NREM EEG power spectra. (**I**) Average REM EEG power spectra. (**J**) Average REM EEG power spectra normalized by the respective wake power spectra. (**K**) Average activity during baseline in 1 h bins. Error bars depict s.e.m. F-value for interaction between factors ‘time’ and ‘strain’ is in the panel. Degrees of freedom are 23, 276. (**L**) Frequency time histogram (71 bins) of activity during baseline. Curves depict group averages, shaded areas depict s.e.m. F for interaction of factors ‘activity level’ and ‘genotype’ (permutation ANOVA) is indicated in the panel. Degrees of freedom are 70, 840.

Figure 3 depicts sleep-related characteristics of S4 and S4-Disc1 mice during 24 h of undisturbed baseline. There were no statistically significant differences in amount of time spent in any of the three analyzed vigilance states (Fig. 3A), average bout durations (Fig. 3B), or bout numbers (Fig. 3C). Group means for vigilance states were 44.8 ± 1.0% wake, 49.9 ± 1.0% NREM, 5.3 ± 0.4% REM for S4, 46.4 ± 1.0% wake, 48.2 ± 0.9% NREM, 5.6 ± 0.16% REM for S4-Disc1. Group means for bout durations were 4.3 ± 0.3 min wake, 2.0 ± 0.1 min NREM, 1.2 ± 0.05 min REM for S4, 4.7 ± 0.2 min wake, 1.9 ± 0.1 min NREM, 1.2 ± 0.06 min REM for S4-Disc1. Group means for bout numbers were 143.0 ± 8.7 wake, 366.6 ± 19.3 NREM, 65.5 ± 6.0 REM for S4, 132.3 ± 4.1 wake, 364.0 ± 23.1 NREM, 66.0 ± 4.4 REM for S4-Disc1.

After we could not detect differences, we calculated the 90% confidence interval of the differences of the means. This is equivalent to the two one-sided test procedure^28^. We can conclude at the alpha = 0.05 level that the real difference of the means is not larger in either direction than indicated by this interval. Confidence bounds were for wake time (percentage points) −4.1, 1.2; NREM time −0.84, 4.3; REM time −1.2, 0.65; for wake bout durations −1.0, 0.25 min; NREM bout durations −0.22, 0.34 min; REM bout durations −0.19, 0.08 min; for wake bout number −8.5, 29.8; NREM bout number −50.7, 55.9; REM bout number −14.6, 13.6. The distribution of sleep time across the 24 h period did not differ between the genotypes (Fig. 3D–F; no interaction between factors “Zeitgeber Time” and “genotype”).

Averaged EEG power spectra did not differ for wake (Fig. 3G), NREM (Fig. 3H), or REM (Fig. 3I). REM spectra normalized by the corresponding wake spectra did not differ between the genotypes, indicating that similar changes in brain activity occur when either group switches between these states (Fig. 3J). Both genotypes showed higher locomotor activity during lights-off, which is expected for a nocturnal species (Fig. 3K). Correspondingly, ANOVA revealed a main effect for the factor ‘Zeitgeber Time’ (F_23,276_ = 5.65, p < 0.001). However, no main effect was found for the factor ‘genotype’ (F_1,12_ = 0.01, p = 0.911) and there was no significant interaction (F_23,276_ = 0.71, p = 0.834), suggesting that diurnal activity patterns did not differ between the mouse lines. A more fine-grained analysis of locomotor activity using a histogram plot also did not reveal any differences between the genotypes (Fig. 3L).

**Figure 4:**
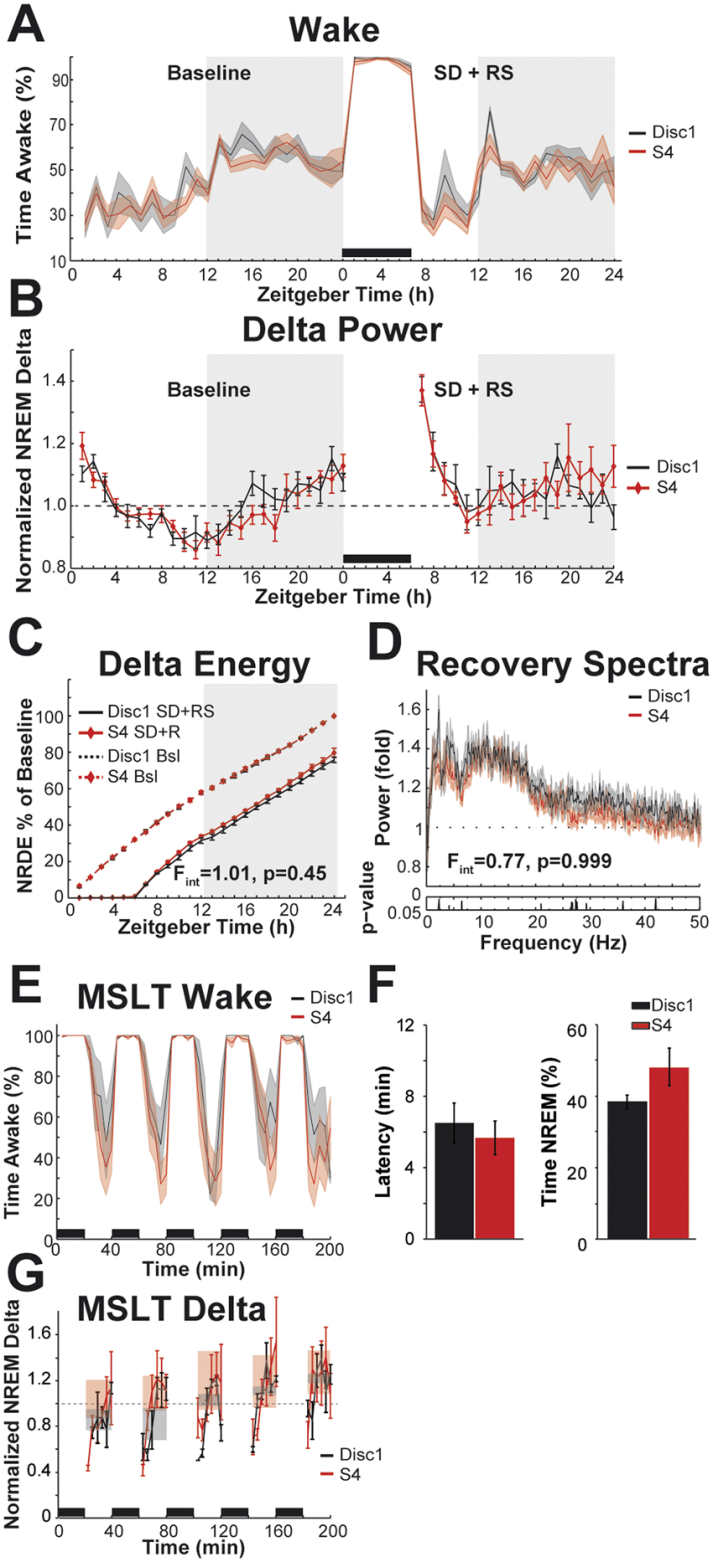
Responses to sleep homeostatic challenges in S4 and S4-Disc1 mice. This figure directly parallels the comparisons made between S4 and B6 in Fig. 2. (**A**) Wakefulness in hourly bins during undisturbed baseline, sleep deprivation, and recovery opportunity. Black bar at bottom indicates sleep deprivation. Shaded background indicates lights-off. Curves depict group means and s.e.m. The baseline values are replotted from Fig. 3D. SD = sleep deprivation, RS = recovery sleep. (**B**) NREM delta power in hourly bins during baseline and recovery sleep opportunity. Black bar at bottom indicates sleep deprivation. Shaded background indicates lights-off. Curves depict group means and s.e.m. (**C**) Cumulative NREM delta energy over 24 h. Dashed curves depict baseline values, solid curves depict values during sleep deprivation and recovery sleep. Curves depict mean values and s.e.m. F-value for interaction of factors ‘time’ and ‘strain’ for the sleep deprivation + recovery days is indicated in the panel. Degrees of freedom are 23, 276. Bsl = baseline. (**D**) NREM EEG power spectra of the first 2 h of RS normalized by the corresponding 2 h of baseline NREM (ZT7 + 8). Curves depict group averages and s.e.m. F-value for interaction of factors ‘frequency’ and ‘genotype’ (permutation ANOVA) is indicated at bottom. The degrees of freedom are 409 and 4908. The p-values for post hoc uncorrected bin-by-bin t-tests are indicated below the spectra. (**E**) Wake time during the multiple sleep latency test. Curves depict group means and s.e.m. Black bars at bottom indicate the five consecutive 20 min sleep deprivations. (**F**) Sleep latency and time spent in NREM during the 5 nap opportunities of the MSLT. (**G**) NREM delta power during the nap opportunities of the MSLT, normalized by the average baseline values. Curves depict group means in 200 s time bins. Error bars depict s.e.m. Shaded rectangles depict group means ± s.e.m. for a complete nap opportunity.

Analyses of sleep homeostasis (Fig. 4) also did not recapitulate any of the differences detected between the background strains. There was no difference between S4 and S4-Disc1 in the time spent asleep in the first 2 h of recovery sleep opportunity following 6 h of sleep deprivation (71.7 ± 2.8 and 69.9 ± 2.7, respectively; T_12_ = 0.434, p = 0.67; Fig. 4A). 90% confidence bounds for the difference of the means were −8.9, 5.4. Figure 4B depicts NREM delta power during baseline and following 6 h of sleep deprivation, both normalized to the average baseline values. The amplitudes of the undisturbed diurnal oscillations did not differ between S4 and S4-Disc1 strains (0.49 ± 0.03 and 0.40 ± 0.04, respectively; T_12_ = 1.75, p = 0.11). 90% confidence bounds for the differences of the mean were −0.002, 0.18. Analysis of NRDE detected no interaction of factors ‘Zeitgeber Time’ and ‘genotype’ (F_23,276_ = 1.01, p = 0.45; Fig. 4C). Analysis of NREM spectra during the first 2 h of recovery sleep opportunity also detected no differences in additional EEG frequencies (Fig. 4D), implicating that the EEG response to acute sleep pressure was identical for mice with either version of the *Disc1* gene. In the murine multiple sleep latency test, we found no difference of latency to sleep between S4 and S4-Disc1 mice (5.7 ± 1.0 min and 6.5 ± 1.1 min, respectively; T_12_ = 0.539, p = 0.60; Fig. 4E,F). 90% confidence bounds of the difference of the mean were −3.4, 1.8. The NREM delta power during the MSLT did not differ, either (Fig. 4G). For the mean values per nap opportunity, there was no main effect for ‘genotype’ (F_1,10_ = 0.32, p = 0.58). However, there was a main effect for ‘nap opportunity’ (F_4,40_ = 7.35) with no interaction (F_4,40_ = 1.94, p = 0.122), suggesting that there was a net accumulation of sleep pressure during this task.

## Discussion

We found several differences in EEG, sleep, and sleep homeostasis between the inbred mouse strains, S4 and B6N. None of these differences persisted in a comparison of S4 and S4-Disc1 mice, which argues against the natural *Disc1* deletion altering sleep regulation. We will first discuss the differences between the strains and second the apparent absence of a sleep phenotype linked to the *Disc1* deletion.

The present study is, to our knowledge, the first description of the sleep phenotype of the S4 mouse strain. Although another 129 substrain was included in a comprehensive description of sleep and EEG phenotypes of different inbred mouse strains from a series of reports by Franken *et al*.^22, 23, 26^, that line 129P2, (formerly 129/Ola, hereafter P2) is very different from S4. The two strains were separated in 1948 and contamination of the P2 line appeared to occur sometime after 1977^24^. This is evidenced by a recent large-scale genomics study. Among 17 strains compared to the C57Bl/6J reference genome (hereafter B6J, also studied by Franken), S4 was not included but two closely related strains were, 129S1 and 129S5, as well as the B6N strain we studied and P2 studied by Franken. The number of mouse strain specific SNPs for each strain were B6N = 1,488; 129S1 = 1,489; 129S5 = 1,991; P2 = 23,677^29^. The large number of SNPs specific to P2 indicates it is very genetically different from B6 and 129S substrains and that the 129S substrains are very similar to each other.

It is well known that characteristics of EEG are heritable^30, 31^ and consequently differ systematically between diverse inbred mouse strains^23^. As expected, we found that the EEG of S4 mice was clearly distinguishable from B6N mice in all vigilance states. Most prominently, the EEG power spectra of S4 mice showed lower relative delta power in NREM, and higher relative theta power in wake. Wake theta power correlates with certain behaviors, such as exploration. However, amplitude and peak frequency of the theta peak can also differ between inbred mouse strains when behavior type is controlled^23^. Either factor might account for the difference in wake theta in this study.

Diurnal oscillation of NREM delta power was less pronounced in S4 than in B6N, although a normal rebound following sleep deprivation excluded a deficit in delta power generation. In combination with our finding that their sleep/wake architecture tended to be more fragmented (more but shorter bouts), it can be speculated that S4 mice tended to respond to sleep pressure with an increase of sleep propensity rather than delta power, and thus might have entered sleep before much sleep pressure had accrued during the baseline recording, preventing further build up. This would be in agreement with the quicker sleep onset in the multiple sleep latency test.

Although their work also included a comparison between B6 and 129 substrains, direct comparisons of Franken *et al*.‘s work and ours should be made cautiously for several reasons. First, the B6 mice were from two different substrains; B6J in Franken *et al*.‘s work, B6N in ours. Despite their genetic similarity e.g. refs 32 and 33 B6N and B6J substrains show differences in a number of behavioral tests, although video-based analysis shows their rest/activity behavior to be indistinguishable at the relevant age^34^. Second, our study used a different EEG electrode configuration, which affects the frequency contributions of the recorded signal. Third, the specific tests performed were not identical. Nonetheless, many relative changes can be compared between studies.

Despite the subtle genetic and behavioral differences cited above, the phenotypes of B6N in our study were very similar to that of B6J in Franken *et al*.’s reports. Similarly, many of the qualitative differences between S4 and B6N mice in our study were also reported for P2 and B6J mice though we also noted some differences. Resembling our S4 data, P2 mice showed decreased NREM delta power and higher (although not significantly) sigma power (11–15 Hz) during 24 h baseline, as well as more time spent in REM than B6^23^. Franken *et al*.^26^ noted an “additional rest period” in B6J but not P2 during the second half of the lights-off period. This resembles the curves of wake and NREM in our B6N and S4 groups (Fig. 1D,E). However, in contrast to S4 in our study, P2 mice did not show a blunted oscillation of NREM delta power. Franken *et al*. also did not detect differences between B6J and P2 in the rate at which lost NRDE is regained^26^, in contrast to our findings in S4. However, to test if delta power kinetics truly differ between S4 and P2, a direct comparison with identical electrode configurations would be required.

The major goal of this study was to evaluate a potential effect for the widespread *Disc1* deletion on sleep. Since we hypothesized that the *Disc1* deletion affects sleep regulation, rather than moving the deleted *Disc1* gene into the B6 background we chose to move the wild-type *Disc1* gene into the S4 background because, in addition to testing the hypothesis, the resulting line would be a useful resource for correcting this deficit. Unexpectedly, none of the sleep or EEG characteristics of S4 mice were altered when we replaced the deleted *Disc1* allele with the full-length version. Given the small sample size of our study, we cannot exclude that the *Disc1* deletion causes smaller effects, which would require a larger study to detect. However, our experimental setup robustly detected differences of sleep and EEG in the background strains, B6N versus S4, but not in the S4 versus S4-Disc1 comparison. We conclude that it is unlikely that the differences between S4 and B6N mice were caused by the *Disc1-*deletion mutation present in S4 and other strains. It is a theoretical possibility that S4 carries additional mutations in a putative *Disc1* dependent, sleep-related pathway. This would make the presence of full-length *Disc1* insufficient to rescue the phenotype. An experiment conversely to ours, where the deleted allele is transferred to a B6 background, would be able to control for this possibility.

Another caveat with this study is that a region of genome flanking the *Disc1* allele in S4-Disc1 mice was from the B6N genetic background and it is possible these linked genes, some of which will have differences from their S4 counterpart, could have resulted in the lack of a detectable phenotypic difference between S4 and S4-Disc1 mice. However, if our conclusion that the *Disc1* deletion does not affect sleep is false, the simplest (although admittedly still complicated) explanation for the lack of a phenotypic difference being due specifically to the presence of a linked B6 derived gene is that the linked B6 gene would make the B6 *Disc1* gene function like the S4 *Disc1* gene when in the S4 but not the B6 background. This could be tested by creating both a new knock-in mouse line that corrects the deletion in inbred S4 mice and one that creates the same 25 base pair *Disc1* deletion in an inbred B6 background. However, since our experiments did not detect differences in congenic mice, it is very unlikely that a knock-in approach will reveal differences either.

Although the purpose of our experiment was to test if the *Disc1* deletion affects sleep, it might be tempting to speculate based on ours and others work if there is a role for wild-type *Disc1* in sleep. Some isoforms of *Disc1* are still expressed in mice carrying the deletion^16^. In contrast to the lack of phenotype in our study, the same mutation affected cognitive function and neuronal development on C57BL/6J or mixed C57BL/6J;129S1/SvImJ backgrounds^17–19^, indicating the deleted *Disc1* gene is not equivalent to the wild-type gene. It is possible that the remaining isoforms are sufficient for retaining a putative sleep-regulatory function, but not other functions of *Disc1*. Alternatively, *Disc1* might not be involved in sleep regulation after all. Indication for a putative role of *Disc1* for sleep comes from two recent transgenic studies, one in Drosophila^12^, the other in mouse^11^. In mouse, the authors expressed the human *DISC1* under the promoter of alpha-CaMKII with a random integration approach. While such models are useful, several potential confounds have to be considered, as we have discussed in detail elsewhere^35^, and briefly summarize as follows. First, random integration of transgenes can produce off-target effects by disrupting other genes at the insertion sites. Second, the location of the transgene’s insertion influences the cell type-specificity of its expression pattern, as does the use of a heterologous promoter. Effects of the gene product in cell types where it would not naturally be expressed may result in a phenotype that does not reflect the physiological function of the transgene. Moreover, expression of both native and exogenous DISC1 protein might also produce effects by non-physiological mechanisms, such as aggregation of excess protein. Indeed, the DISC1 protein appears to be prone to aggregation^36^. Some of these gain-of-function mechanisms might indeed link *DISC1* to brain functions in human pathologies^36^, possibly including disturbed sleep. However, this could be the case also in the absence of a physiological role of *DISC1* in sleep-regulation. Likewise, our study was focused on a natural deletion mutation encoding a premature stop codon that appears to be partially skipped over by alternative splicing^16^. These caveats must be addressed before a firm conclusion about a putative role of wild-type *Disc1* for sleep regulation is reached. A mouse line in which no *Disc1* gene product is made could more conclusively address whether there is a role for wild-type *Disc1* in sleep.

We can conclude, however, that the natural deletion mutation of *Disc1* found in 129 and other widely used mouse strains does not noticeably affect sleep regulation. This is important for the field of sleep research. Many relevant transgenic mouse lines are created using stem cells derived from affected lines, including complete or partial knockout mice e.g. refs 37–39, cre lines for sleep-related neurotransmitters e.g. refs 40–43 and reporter lines for selective manipulation of sleep-related cell types e.g. refs 44 and 45. It has been cautioned that the possible presence of the *Disc1* deletion mutation in such mice could confound the study of behaviors that are affected by this mutation^13^. Our finding suggests that this is no reason for concern for sleep research.

## Methods

### Breeding of S4-Disc1 mice

S4 mice congenic for the wild-type (WT) *Disc1* allele (S4-Disc1) were derived by nine rounds of backcrossing of the *Disc1* allele derived from B6N into the S4 background aided by a PCR assay developed previously^13^. Briefly, PCR with 36 cycles of 94 °C 20 sec, 54 °C 60 sec, 72 °C 40 sec using two primers (Forward-5′-GCTGTGACCTGATGGCACT and Reverse-5′-GCAAAGTCACCTCAATAACCA) produced a 196 base pair product for the WT (B6N derived) allele and a 171 base pair product for the deleted (S4 derived) allele. Early in this process the WT allele was passed through the female germline directly for two generations (2 and 3), ensuring that the developing line carried the S4 Y chromosome. Subsequently, the WT allele was passed through the male germline directly for 5 generations (4 through 8), ensuring that the X chromosomes were S4 derived and essentially all B6 mitochondria were diluted out. At generation four a SNP (single nucleotide polymorphism) analysis measuring 385 loci identified a male that was 95% S4, compared to the 93.75% predicted by chance. The lineage was again analyzed at generation seven with a 1417 SNP panel and a male that was 99.58% S4 was identified, compared to the predicted 99.375%. Since the allele was subsequently passed through S4 two more times, the resulting mice were theoretically 99.895% S4. This is possibly an overestimate but we can safely conclude the mice are >99.6% S4.

### Experimental mice

For the first set of experiments we used male mice of strains C57BL/6NCrl (N = 8, age 28.9 ± 1.9 weeks, mean ± standard deviation), and S4 (N = 6, age 27.7 ± 1.1 weeks). For the second set of experiments, we used male mice of strains S4-Disc1 (N = 6, age 26.6 ± 1.5 weeks) and S4 (N = 8, age 25.0 ± 3.1 weeks). All mice were bred in house.

Mice were single housed in cages, which were placed in ventilated cabinets. The cabinets were not temperature- or humidity controlled, but the rooms were kept at 23 ± 1 °C and 45–55% humidity. Mice were provided food and water ad libitum, bedding, and nesting material. Light conditions were 12h/12h of lights on/lights off. In an effort to standardize recording conditions in the lab, the light sources were changed between the two sets of experiments. In the first set, cabinets were illuminated by LEDs, providing ~30 lux at cage level, and experiments were performed in spring. In the second set, cabinets were illuminated by fluorescent tubes, providing ~100 lux at cage level, and experiments were performed in late summer. Experiments were approved by the State Office of North Rhine-Westphalia, Department of Nature, Environment and Consumerism (LANUV NRW). Experiments were carried out in accordance with the relevant guidelines and regulations. All measures were taken to minimize the number of animals used, as well as to maximally reduce their discomfort and pain.

### Surgical Procedures

Mice were implanted with F20-EET transmitters (channel bandwith 1–50 Hz, Data Sciences International, St. Paul, MN) under isoflurane anesthesia. Transmitters were placed intraperitoneally in the first set of experiments and subcutaneously in the second set. The change of transmitter placement was part of standardizing the temperature recordings using this setup, which were used for unrelated studies. EEG leads were placed epidurally above the right parietal cortex (AP-0.5, ML 2.5 mm from bregma, negative lead) and above the cerebellum (center of interparietal bone, positive lead). EMG leads were anchored in the neck muscles. Mice were allowed at least two weeks of recovery before recordings.

### Data acquisition and analysis

Sleep scoring and analysis was done as reported before^38, 46, 47^. EEG, EMG, and signal strength (as proxy for locomotor activity) were recorded via telemetry using DQ ART software (Data Sciences International). Sampling frequencies were 500 Hz, low-pass filter cut offs were 100 Hz (in addition to the 1 Hz high pass and 50 Hz low pass antialiasing filtering built in the transmitter). EEG and EMG recordings were scored in 10 s epochs as wake, rapid eye movement sleep (REM), or non-rapid eye movement sleep (NREM) by expert scorers who examined the recordings visually using NeuroScore 3.0 software (Data Sciences International). To calculate bout durations, a bout was defined as consisting of a minimum of two consecutive epochs of a given state and ending with a single state change epoch. Latency to sleep was the time from start of a nap opportunity to the first occurrence of a NREM bout. EEG spectra were analyzed with a fast Fourier transform algorithm using a Hanning Window without overlap (NeuroScore) on all epochs without artifact. For direct comparisons of EEG power spectra, power was expressed as relative power, i.e. each frequency bin (0.122 Hz) was divided by the sum of the values between 0 and 50 Hz. Normalized NREM delta power was calculated as the raw power between 1–4 Hz (i.e. summed values of the respective frequency bins) of any epoch scored as NREM, divided by the average NREM delta power of the 24 h baseline recording. “Signal strength” is a measure that reflects position of the transmitter relative to the receiver antennae. It does not influence quality or amplitude of any of the transmitted data (i.e. EEG/EMG). The manufacturer DSI uses changes of signal strength as the mouse moves across the receiver plate to derive an “activity count”, which reflects amount and speed of motion per time. This is a semi-quantitative measure, which gives results comparable to the established beam break method^48^, and has been used to compare the overall activity between groups of mice^49^. To obtain a continuous rather than a discrete measure (as in DSI’s “activity count”), we expressed activity as the standard deviation of the signal strength values for each epoch, which gives a redundant (but continuous) measure.

### Sleep deprivation

To probe the homeostatic response to sleep loss, mice were sleep deprived for six hours. Sleep deprivation started at lights on, i.e. Zeitgeber Time (ZT) 0, followed by 18 h of undisturbed recovery sleep opportunity. When a mouse assumed pre-sleep behavior, e.g. lowering the head to the floor, sleep was prohibited by tapping the cage or, if necessary, touching fur or whiskers with a soft brush.

To probe sleep propensity, mice were subjected to the murine multiple sleep latency test MSLT^27^. Starting six hours after lights on (ZT6), mice were sleep deprived for 20 min as described above. This was followed by a 20 minutes nap opportunity. This cycle was directly repeated 5 times.

### Statistics

Statistical tests were performed using MATLAB (Mathworks, Natick, MA) and SPSS (IBM, Armonk, NY). For comparisons of EEG power spectra, we first performed two-way permutation ANOVA^50^ with 5000 iterations with factors ‘frequency bin’ and ‘mouse line’. If interactions were found, the source of the interaction was evaluated through bin-by-bin uncorrected t-tests between the groups. Only changes that affected a range of neighboring frequency bins were considered potentially meaningful. The same analysis was done for activity histograms, with factors ‘activity’ and ‘mouse line’. If not indicated otherwise, reported values are group means ± standard error of the mean.

## Electronic supplementary material

Supplementary Information
