# Supplementary material for: The natural Disc1-deletion present in several inbred mouse strains does not affect sleep
Source: Sci Rep. 2017 Jul 18;7:5665. doi: 10.1038/s41598-017-06015-3 (PMC5515846; doi:10.1038/s41598-017-06015-3)
Supplement: Supplementary file 1 — Supplementary Information [file 41598_2017_6015_MOESM1_ESM.pdf]

The natural *Disc1*-deletion present in several inbred mouse strains does not affect sleep

Abbreviated title: *Disc1*-deletion does not affect sleep

Lars Dittrich, PhD; Alessandro Petese, MSc; Walker S. Jackson, PhD*

Deutsches Zentrum für Neurodegenerative Erkrankungen (DZNE), Sigmund-Freud-Str. 27

53127 Bonn, Germany

Supplementary Information

Supplementary Figure S1. Raw spectra and activity counts. To aid interpretation of differences we found when comparing relative spectra in Figure 1 G-I, the non-normalized data are provided here. A) Raw wake spectra, B) raw NREM spectra, C) raw REM spectra. Curves depict group averages, shaded areas depict s.e.m. Interactions of factors 'frequency' and 'strain' (permutation ANOVA) are indicated for each panel. The degrees of freedom are 409 and 4908 for all interactions. D) Average activity counts for 24 h baseline periods. The proprietary activity count measure provided by the recording system was used. B6 and Disc1 groups were compared to the corresponding S4 groups. *p<0.05, n.s. = not significant, t-test. E) Activity counts over the 24 h baseline period in 1 h bins for B6 and the corresponding S4 group. Repeated measures ANOVA showed a main effect for strain ($F_{1,12}$ = 6.9, p = 0.02), but no interaction of strain and Zeitgeber Time ($F_{23,276}$ = 1.2, p = 0.24). F) Activity counts over 24 h baseline for Disc1 and the corresponding S4 group. Repeated measures ANOVA showed no significant effects for genotype or genotype by Zeitgeber Time interaction.

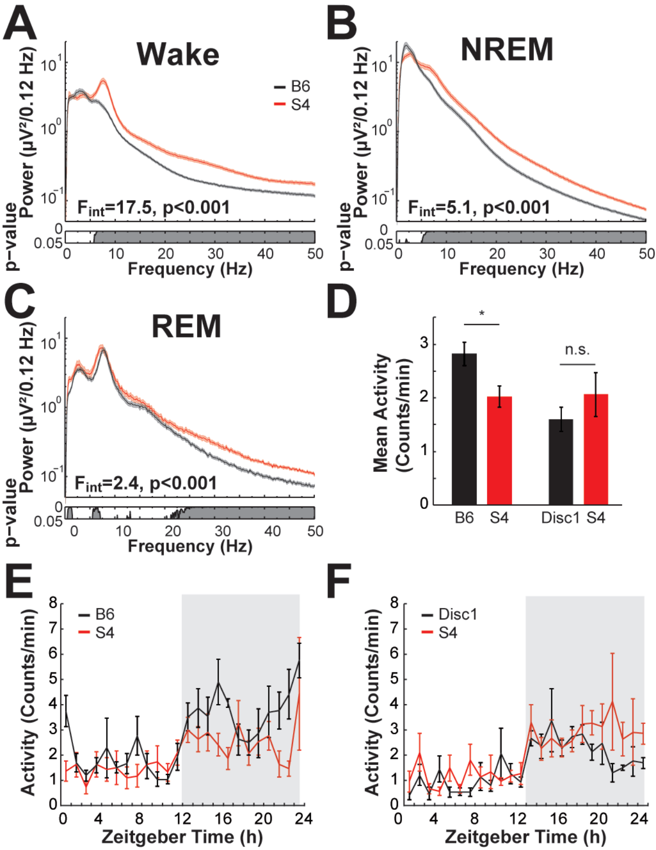
A
Wake
B6
S4
Power (μV²/0.12 Hz)
p−value
Fint=17.5, p<0.001
Frequency (Hz)
B
NREM
Fint=5.1, p<0.001
C
REM
Fint=2.4, p<0.001
D
Mean Activity (Counts/min)
*
n.s.
B6
S4
Disc1
S4
E
Activity (Counts/min)
Zeitgeber Time (h)
F
Disc1
S4
